# Change of Immunoglobulin Superfamily Member 4 Gene Expression in the Digestive System after Swimming Exercise in BALB/c Mice

**Published:** 2018-11

**Authors:** Eun-Ju CHOI, Wi-Young SO

**Affiliations:** 1. Dept. of Physical Education, Daegu Catholic University, Gyeongsan, Korea; 2. Sports and Health Care Major, Korea National University of Transportation, Chungju-si, Korea

## Dear Editor-in-Chief

Immunoglobulin superfamily 4 (IGSF4) is a member of the intercellular adhesion molecule family (1). IGSF4 encodes a transmembrane protein whose extracellular domain shows close homology to the immunoglobulin superfamily cell adhesion molecules, particularly the neural cell adhesion molecules and the prostate tumor-suppressor TSLL2/IGSF4C (2, 3). Thus, IGSF4 is related to immune function (1,4).

Silencing of IGSF4 is frequently observed in solid tumors including lung, prostate, pancreatic, gastric, breast, nasopharyngeal, and cervical cancers, as well as nasal natural killer T cell lymphoma (2). Moreover, IGSF4 is a stress-responsive gene capable of inducing apoptosis (2). However, a link between IGSF4 and exercise in the digestive system was not suggested in that study or elsewhere.

The purpose of this study was to determine whether exercise differentially affected IGSF4 gene expression in the digestive system and could play an important role in its induction in small intestine and colon in a mouse model.

Eight-week-old male BALB/c mice were divided randomly into control (n=10) and swimming exercise (n=10) groups. Swimming was conducted in a plastic pool 60 cm high and 120 cm in diameter for 30 min, 5 days/week for 4 wk. The water temperature of the pool was set to 35–37 °C. Water was filled to more than 45 cm to assure mice could not touch the bottom during swimming.

Total RNA was isolated from cells or homogenized tissues of BALB/c mice with TRIZOL re-agent and reverse transcribed using RT-PreMix (Enzynomics, Daejeon, Korea). mRNA levels were measured using the real-time polymerase chain reaction with the following primers (the respective forward and reverse pairs are indicated): mouse IGSF4, 5′-CAG TAT AAA CCG CAA GTG CA-3′ and 5′-GCG GTA AGT ACC GTT ATC TG- 3′; mouse GAPDH, 5′-GCA CAG TCA AGG CCG AGA AT- 3′ and 5′-GC CTT CTC CAT GGT GGT GAA-3′. The amplification profile was composed of denaturation at 94 °C for 30 sec, annealing at 60 °C for 20 sec, and extension at 72 °C for 40 sec. The 30 amplification cycles were preceded by denaturation at 72 °C for 7 min. Amplification was performed in the DNA Engine Opticon system (Applied Bio-systems, Waltham, MA, USA). Fluorescence was detected continuously in a total volume of 10 μL containing 1 μL cDNA/control and gene-specific primers by using SYBR Premix Ex Taq (Takara Bio, Shiga, Japan). mRNA levels of the target genes, relative to *GAPDH*, were normalized by using the following formula: relative mRNA expression =2Δ(ΔCt of target geneΔΔCt of GAPDH), where Ct is the threshold cycle value. In each sample, the expression of the gene being analyzed was normalized to that of *GAPDH* and expressed as the mean mRNA level ± standard deviation. Data were analyzed using AVADIS prophetic version 3.3 software (Strand Genomics, Bangalore, India) and the independent *t-*test was performed using SPSS version 18.0 (IBM, Chicago, IL, USA). Statistical significance was set at *P*<0.05.

**Fig. 1:**
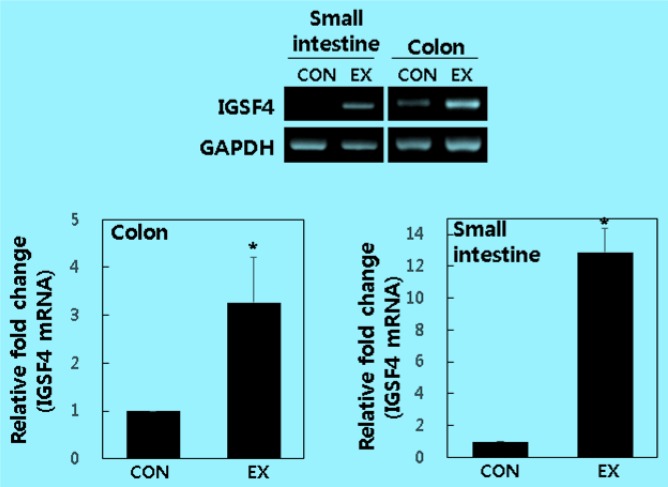
mRNA levels of IGSF4 in small intestine and colon tissue of BALB/c mice with and without exercise. Data are expressed as means ± standard deviation relative to GAPDH mRNA (n=10). *Significantly different from control (*P*<0.05)

Fig. 1 shows the small intestine and colon tissue levels of IGSF4 mRNA after swimming exercise in BALB/c mice.

The fold increases of IGSF4 in small intestine and colon following swimming exercise relative to the control were 3.27±0.95 and 12.87±1.53, respectively. IGSF4 expression of both small intestine and colon in swimming exercise was significantly increased compared with control (*P*<0.05).

We demonstrated the induction of IGSF4 gene expression by exercise in the digestive system. Exercise is likely to benefit the immune system in these tissues.
